# Editorial comment from Dr Yuasa to Pathological complete response after nivolumab therapy following angiogenesis inhibitors in a patient with metastatic renal cell carcinoma

**DOI:** 10.1002/iju5.12228

**Published:** 2020-10-07

**Authors:** Takeshi Yuasa

**Affiliations:** ^1^ Department of Urology Cancer Institute Hospital Japanese Foundation for Cancer Research Ariake Tokyo Japan

The indication and the role of metastasectomy for patients with metastatic renal cell cancer (RCC) remains unclear.^1^ Various retrospective studies have reported the survival benefit resulting from the aggressive complete surgical removal in selected patients.^2^ It is also true, however, that there is always selection bias and the results have to be interpreted with caution.^1^ To date, there have been no randomized controlled trials that have evaluated the clinical benefit of the metastasectomy.^1^ The European Society for Medical Oncology recommends that patient selection should be discussed within a multidisciplinary team.^1^, ^3^ The patients with good performance status, solitary or oligometastases, metachronous disease with disease‐free interval >2 years, the absence of progression on systemic therapy, low or intermediate Fuhrman grade, and complete resection may be good candidates and have been associated with favorable outcome after metastasectomy.^1^, ^3^

In this issue of *IJU Case Reports*, Hagimoto *et al*. reported a case of metastatic RCC who achieved a durable response using nivolumab as the fifth‐line therapy following the first‐line interferon‐alpha, the second‐line sunitinib, the third‐line axitinib, and the fourth‐line everolimus.^4^ After initiation of nivolumab therapy, the patient’s symptoms and laboratory results dramatically improved.^4^ The lung metastasis diminished, achieving a complete response (CR), and the adrenal gland metastasis shrunk in size.^4^ Nivolumab therapy was discontinued due to the development of grade 2 acute kidney injury and grade 2 maculopapular rash, which were clinically diagnosed as immune‐related adverse events.^4^ The patient underwent surgical removal of the residual shrinking adrenal gland metastasis, which demonstrated pathological CR after the surgery.^4^ No local recurrence or metastasis has been observed for >20 months postoperatively.^4^

The patient seemed to be successfully treated with sequential therapy using an interferon, targeted agents, and an immune checkpoint inhibitor followed by metastasectomy. Pathological CR sounds good, but is it accurate? Based on these results, this metastasectomy might be considered unnecessary because there were no cancer cells. I believe that the potential benefits of metastasectomy are as follows: (i) a patient can discontinue his or her medical therapy at least for a certain period until a recurrence is diagnosed and (ii) when most other metastatic lesions shrink but only one or two lesions grow (mixed response), surgical removal of only the growing lesions can achieve surgical CR. Others also described the potential benefit of the surgical removal of the growing tumor.^5^ In this case presented by Hagimoto *et al*., the medical therapy had already been discontinued before surgical removal. Moreover, the adrenal metastasis was decreasing in size. Observation without medical and surgical therapies might be a treatment option. Although it may appear that I am being completely critical of this choice, this is not the case. Treatment selection is always difficult in clinical practice, and further investigation is necessary to clarify this important clinical theme.

## Conflict of interest

The author declares no conflict of interest.
